# Blunted Social Reward Responsiveness Moderates the Effect of Lifetime Social Stress Exposure on Depressive Symptoms

**DOI:** 10.3389/fnbeh.2019.00178

**Published:** 2019-08-07

**Authors:** Samantha Pegg, Paige Ethridge, Grant S. Shields, George M. Slavich, Anna Weinberg, Autumn Kujawa

**Affiliations:** ^1^Department of Psychology and Human Development, Vanderbilt University, Nashville, TN, United States; ^2^Department of Psychology, McGill University, Montreal, QC, Canada; ^3^Center for Mind and Brain, University of California, Davis, Davis, CA, United States; ^4^Department of Psychiatry and Biobehavioral Sciences, Cousins Center for Psychoneuroimmunology, University of California, Los Angeles, Los Angeles, CA, United States

**Keywords:** reward responsiveness, social reward, life stress, neurophysiology, event-related potentials, electroencephalogram, depression

## Abstract

Exposure to social stress is a well-established risk factor for the development and recurrence of depression. Reduced neural responsiveness to monetary reward has been associated with greater symptoms following stress exposure. However, it remains unclear whether reduced reward responsiveness serves as a mediator or moderator of the effects of stress on internalizing symptoms or whether similar patterns emerge with responses to social reward. We addressed this issue by measuring lifetime stress exposure and event-related potentials (ERPs) to social reward in 231 emerging adults (*M* = 18.16, *SD* = 0.41 years old). Participants completed the Stress and Adversity Inventory (STRAIN) to assess severity of lifetime stressors and self-report measures of current internalizing symptoms. In addition, participants completed the Island Getaway task in which the reward positivity (RewP) ERP was recorded in response to social acceptance, adjusting for responses to rejection (RewP residual). In this task, participants vote to accept or reject peers and receive reward/acceptance and rejection feedback. Stressors were divided into social and non-social stress severity scores. Analyses were conducted to test social reward responsiveness as a mediator or moderator of the effects of social and non-social stress on internalizing symptoms. Both social and non-social stress exposure over the life course predicted symptoms of depression (*p*s < 0.001) and social anxiety (*p*s < 0.002). The effect of social stress on depression was moderated by the residual RewP to social reward, adjusting for responses to social rejection (*p* =0.024), such that greater lifetime social stress exposure and a relatively blunted RewP to social reward were associated with greater depressive symptoms. Social reward responsiveness did not mediate effects of stress on internalizing symptoms. Reduced processing of social reward may be a vulnerability for depression that increases risk for symptoms following exposure to social stress. Blunted social reward responsiveness appears to be a relatively unique vulnerability for depression, rather than social anxiety. Results support the utility of ERP measures in measuring individual differences in social reward processing that can be applied to better understand neural processes involved in the development of depression, and highlight the importance of considering specific dimensions of stressful life experiences.

## Introduction

Life stress exposure is a well-established risk factor for depression (Kendler et al., 1999; Hammen, 2005; Kessler et al., 2010; Slavich, 2016). Experiencing more stressful life events in childhood is associated with increased risk for both recent and lifetime history of depressive disorders (Chapman et al., 2004). In fact, exposure to stressful life events during the past year is a strong risk factor for and precursor to the development of major depression (Kendler et al., 2002, 2006). In this context, interpersonal stress has been shown to have particularly strong effects on depression risk (Hammen, 2009). For example, depressive episodes have been related to humiliating life events, characterized by situations in which a person is devalued in an important role (Kendler et al., 2003). Additionally, individuals diagnosed with major depressive disorder (MDD) who experienced a severe targeted rejection life event prior to onset have been found to develop depression three times faster than persons experiencing other types of severe, pre-onset life stress (Slavich et al., 2009).

Despite these strong associations between exposure to life stress and the development of depression, many people who experience even major life stressors during their lives do not develop depression. Therefore, there is a need to identify processes that make some people more likely than others to develop depression following exposure to stress. These vulnerabilities likely depend in part on genes and brain function. For example, genetic factors related to neural response to rejection have been shown to differentiate individuals diagnosed with MDD from those who are not following a targeted rejection stressful event (Slavich et al., 2014). In terms of brain function, neuroscience research has been shown to have the potential to elucidate alterations in brain function that make some people more susceptible to develop depression in response to stress (Kujawa and Burkhouse, 2017). Overactivation of threat circuits, including the amygdala, has been shown to predict response to stress, including stress related to natural disasters, terrorist attacks, and more typical life stress (McLaughlin et al., 2014; Swartz et al., 2015; Kujawa et al., 2016).

There is also growing evidence that deficits in positive valence systems, which include reward responsiveness, play a key role in pathways from stress to depression. For example, one study found that life stress over the past year was associated with low positive affect only in persons with low ventral striatum activity – a key subcortical brain region involved in reward processing and motivation – in response to monetary reward (Nikolova et al., 2012). Additionally, reduced activity in the ventral striatum is related to increased risk for anhedonia in individuals exposed to early life stress (Corral-Frías et al., 2015). These data suggest that low reward responsiveness – typically assessed in response to monetary reward – might be a vulnerability factor that moderates the effects of stress on the emergence of depression.

Other research has suggested a more mechanistic relationship between stress and neural response to reward – namely, that stress may reduce reward responsiveness, which in turn leads to depressive symptoms. For example, some types of early life stress have been associated with reduced striatal activation, which predicts depressive symptoms later in life (Goff et al., 2013; Hanson et al., 2015). In addition to striatal activation, research has examined neurophysiological indicators of activation of reward learning systems such as the reward positivity (RewP), an event-related potential (ERP) enhanced in response to positive feedback and rewards (Holroyd and Coles, 2002, 2008; Carlson et al., 2011). In monetary reward tasks, RewP is associated with activation in brain regions involved in reward processing, including the ventral striatum and medial prefrontal cortex (Carlson et al., 2011). Similar to findings from neuroimaging studies examining brain regions involved in reward processing, research investigating RewP has found that a reduced RewP to monetary rewards prospectively predicts depressive symptoms across childhood and adolescence (Bress et al., 2015; Nelson et al., 2016; Kujawa et al., 2019). Additionally, recent research has shown that RewP to monetary reward measured in childhood interacts with acute stressful events to predict depressive symptoms in early adolescence (Goldstein et al., 2019). However, it remains unclear how this manifests with regard to social reward and to specific types of stressful experiences, as well as the extent to which reduced reward responsiveness as measured by RewP reflects a moderator or mechanism of the effects of stress on depressive symptoms.

Critically, prior reward responsiveness research has primarily focused on monetary reward. Although this work has shown that alterations in reward responsiveness are associated with the development of depressive symptoms (e.g., Kujawa et al., 2019), measuring reward responsiveness only in response to monetary rewards has limitations. For example, individuals vary in the extent to which they value the same amount of money. In addition, laboratory-based monetary reward tasks typically offer relatively small amounts of money, and tasks vary from one another in the amount they offer, which may have an impact on task engagement and reward valuation. Social reward, instead, may be a stronger or more consistent predictor of social behaviors and clinical symptoms (Davey et al., 2008; Forbes and Dahl, 2012; Silk et al., 2012). In addition, alterations in response to social reward may be particularly relevant for examining how different individuals fare under interpersonal stress. For example, individuals at risk for depression may not be as responsive to or less motivated to participate in positive social activities (Setterfield et al., 2016), particularly when they experience stress. However, little is known about the relationship between *social* reward responsiveness, *social* stress exposure, and internalizing symptoms, even though social stress is the strongest psychosocial precipitant of MDD (e.g., Hammen, 2009). Compared to monetary reward, responses to social rewards might be more relevant when considering response to interpersonal experiences and/or predict specific features of depression (e.g., social withdrawal/anhedonia). Additionally, we may be able to better predict response to specific types of stressors by examining relations between distinct types of reward, specific types of stress, and the development of depressive symptoms.

One ERP task that has been developed to examine neural reactivity to social reward is the Island Getaway task (Kujawa et al., 2014). In this game, participants interact with perceived peers and give and receive positive and negative social feedback in the form of votes to stay in or get kicked out of the game across several rounds. This task consistently elicits a RewP enhanced in response to social reward/acceptance feedback, maximal over frontocentral sites, and with similar timing as observed in monetary reward tasks (Ethridge et al., 2017). RewP can be reliably assessed across development (Kujawa et al., 2018), including in response to social reward using the Island Getaway task (Ethridge and Weinberg, 2018). Yet, relatively little is known about the RewP in the context of social reward, including the extent to which social reward responsiveness might serve as a mediator or moderator of the effects of stress on depressive symptoms.

In addition, much of the research on reward responsiveness and stress has focused on subjective experiences of stress, the measurement of which is often confounded with the assessment of depressive symptoms (Slavich, 2019). Measures of stress can also vary in numerous ways, including in how comprehensively they assess stressors, their consideration of chronic vs. acute stressors, the types of stressors assessed (e.g., minor vs. severe stressors), the timeframe assessed, and the frequency and duration of stressor exposure assessed (Epel et al., 2018; Slavich, 2019). The Stress and Adversity Inventory (STRAIN; Slavich and Shields, 2018) was developed to address these issues by providing investigators with a standardized system for assessing lifetime stress exposure across a number of different stressor types (acute vs. chronic), timespans (childhood, adulthood), life domains, and social-psychological characteristics. In the present study, we employed the STRAIN to characterize participants’ total lifetime severity of stressors experienced across these categories.

More specifically, we examined associations between lifetime exposure to social and non-social stressors, neurophysiological response to social reward, and internalizing symptoms in a large sample of emerging adults. We sought to provide a preliminary examination of the utility of social reward responsiveness in understanding links between stress exposure and internalizing symptoms. To extend the existing literature on monetary reward responsiveness, we tested competing theories of the role of reward responsiveness in depression by investigating whether social reward responsiveness moderated (e.g., Nikolova et al., 2012; Corral-Frías et al., 2015) or mediated (e.g., Goff et al., 2013; Hanson et al., 2015) the effects of social and non-social stress on symptoms of depression. In addition, we tested these associations for both social and non-social stress exposure to examine whether interpersonal aspects of reward processing mediate or moderate the effects of social stress specifically. Although we were primarily motivated by models of reward responsiveness in depression, we explored similar models predicting symptoms of social anxiety in order to test whether observed associations were specific to depression or also present for other internalizing symptoms. Social anxiety represents a logical comparison in this context, as social stressors – including problems in peer relationships – have been found to predict both social anxiety and depressive symptoms (La Greca and Harrison, 2005; Starr and Davila, 2008). Alterations in social reward responsiveness could reflect a relatively specific neural process underlying symptoms of depression in particular (e.g., Bress et al., 2015; Nelson et al., 2016) or could underlie both depression and anxiety symptoms more broadly.

## Materials and Methods

### Participants

A total of 268 emerging adults were recruited at the start of their first year of college and completed the Island Getaway task for a larger study examining neural mediators and moderators of the effects of stress on internalizing symptoms. In this larger study, we aimed to recruit up to 100 first-year students per year for 3 years for a total sample size with adequate power to detect generally modest associations between neural and clinical measures. Following written informed consent in accordance with the Declaration of Helsinki, participants completed a series of EEG tasks in a counterbalanced order, the results of which have been previously reported (Ethridge and Weinberg, 2018; Sandre et al., 2019), along with measures of stress exposure and clinical symptoms. Of this sample, 13 were excluded due to a computer error during data collection, 3 for not completing the measure of clinical symptoms, 20 for not completing the STRAIN, and 1 due to excessive noise in EEG data. The final sample thus included 231 emerging adults (*M* = 18.16, *SD* = 0.41 years). Most participants identified as female (71.9%) and Caucasian (51.3%). All study procedures were approved by the McGill University research ethics board. All data exclusions, measures, and conditions have been disclosed in the present manuscript.

### Measures

#### Lifetime Stress Exposure

To assess the frequency and subjective severity of participants’ exposure to different stressors across the life course, individuals completed the STRAIN online (Slavich and Shields, 2018). The STRAIN assesses stressors occurring across several life domains, including: Housing, Education, Work, Treatment/Health, Marital/Partner, Reproduction, Financial, Legal/Crime, Other Relationships, Death, Life Threatening Situation, and Possessions. Participants first respond to introductory questions for stressors in each life domain; then, if a stressor was endorsed, they were asked additional questions about the severity, frequency, timing, and duration of the stressor.

To differentiate lifetime social and non-social stress severity, all items that were related to interpersonal or social situations/interactions (i.e., that had a primary underlying social-psychological characteristic that was social) were binned into the social stress variable. Social items included questions such as, “Have you ever had ongoing arguments with a spouse or partner?”, “Were you ever bullied by other kids at school?”, and “Did moving to college make you lose contact with friends?” All remaining items were binned into the non-social variable. Non-social items included questions such as, “Have you ever looked for a job for at least 6 months?”, “Have you ever been hospitalized because of a health problem?”, and “Have you failed a class or been in danger of failing a class in college?” The resulting lifetime social stress severity composite had 51 total items, and the non-social stress severity composite had 30 items.

#### Internalizing Symptoms

Both depression and social anxiety were investigated in the present study using the Inventory of Depression and Anxiety Symptoms (IDAS), a 99-item, validated measure of current (i.e., past 2 weeks) anxiety and depressive symptoms (Watson et al., 2007). The IDAS is comprised of 10 specific symptom scales, including social anxiety, and broader scales, including dysphoria, which is composed of single items that assess depressed mood, anhedonia, worry, worthlessness, guilt, psychomotor agitation, psychomotor retardation, and hopelessness, as well as two items assessing cognitive problems (Watson et al., 2007). The rating scales range from 1 (*Not at all*) to 5 (*Extremely*). We used the dysphoria subscale to measure depressive symptoms, the primary outcome of interest. We also tested models including social anxiety symptoms to evaluate specificity of these effects for depression vs. internalizing symptoms more broadly.

#### EEG Task

Participants completed the Island Getaway task while EEG data were collected (Kujawa et al., 2014; Ethridge et al., 2017). Task code for prior versions of Island Getaway are available here: http://arfer.net/projects/survivor. In this task, participants were told that they would be playing a “Survivor”-style computer game with other students their age where they would travel along the Hawaiian Islands with co-players, trying to make it to the final island without being voted off along the way. Co-players included 11 confederate peers, whom participants were led to believe were other college students completing the task not necessarily as part of the same experiment or in the same building as the participant. Prior to beginning the task, a photograph was taken for the participant’s game profile picture. They were then told about the overall concept and goal of the game. They first answered several questions to create a profile, including questions about their name, age, hometown, and general interests and reviewed the profile information of their co-players. Hometowns of the co-players included cities in Canada and the United States, usually close to large universities (e.g., Toronto, New York City).

Each round, participants were presented with the profile information of the other players and decided to vote to either accept (i.e., “Keep”) or reject (i.e., “Kick out”) each co-player, while led to believe that co-player was simultaneously voting to accept or reject the participant. Each profile was presented until the participant voted. To make the task more realistic, a statement appeared on the screen saying, “Waiting for [co-player name] to vote…,” if participants voted faster than the simulated voting time assigned to the co-player for that round (based on actual voting speeds from pilot testing). Following the vote, a fixation cross was presented for 1000 ms, followed by feedback indicating how the co-player voted for the participant. A green thumbs up was shown on the screen indicating social reward/acceptance feedback, and a red thumbs down was presented indicating social rejection. Feedback was displayed for 2000 ms. This was followed by a screen that had two scales for participants to rate how much they liked the co-player and how much they thought other people would like the co-player, ranging from 1 (*Not at all*) to 9 (*Extremely*). Participants then saw a blank screen for 1500 ms before the next co-player profile within the round was presented. At the end of each round, participants were shown the picture of the co-player that was voted off during that round. All participants reached the final island at the end of the sixth and final round. Over the course of the 51 trials across the six rounds, participants were presented with roughly equal acceptance and rejection feedback, but ultimately “won” the game without being voted out by peers.

To increase believability, members of study staff acted as though they were in communication with other labs during the study setup and introduced pauses in the experiment to “wait” for other labs to be ready to begin. At the end of the task, prior to being debriefed, participants were asked to verbally indicate whether they believed that the task that they were playing was real in that they were playing against other live players. This was assessed with a 1-item question on a scale from 1 to 5, with higher scores indicating stronger belief in the task. On average, participants reported that they moderately believed that the task was real (*M* = 3.35, *SD* = 1.36), and belief ratings were not correlated with the residual RewP measure obtained from this task (*p* = 0.804).

### EEG Data Collection and Processing

EEG data were recorded with a 32-electrode cap BrainProducts actiCHamp system (Munich, Germany) based on a standard 10/20 layout. Facial electrodes were placed approximately 1 cm above and below the left eye and 1 cm from the outer corners of the eyes to measure electrooculogram (EOG) from eye movements. Bipolar electrodes were referenced to an electrode placed on the back of the neck of the participant. Mastoid references were electrodes TP9 and TP10. Impedances were reduced to approximately 10 kΩ. A 24 bit resolution and sampling rate of 1000 Hz were used to digitize the recordings.

BrainVision Analyzer software (Brain Products, Munich, Germany) was used to process the EEG data. Data were re-referenced to an average of the two mastoids and band-pass filtered with 0.01 and 30 Hz as cutoffs with 24 db/oct slopes. Data were segmented 500 ms prior to and 1000 ms after acceptance/rejection feedback. Ocular correction was conducted using a modification of Gratton’s algorithm (Gratton et al., 1983). Automatic artifact rejection criteria were a voltage step greater than 50.0 μV between sample points, maximum voltage difference of 175.0 μV within trials, and minimum voltage difference of 0.5 μV within 100 ms intervals. Data were then inspected visually to reject any remaining artifacts. Following artifact rejection procedures, participants had on average 26.61 (*SD* = 1.44) trials for the accept condition and 24.06 (*SD* = 1.32) trials for the reject condition at Cz. The 200 ms prior to feedback was set as the baseline.

ERPs were averaged across participants for both acceptance/social reward and rejection/non-reward. ERP components were scored using the time window approach based on visual assessment. To examine RewP, data were extracted between 250 and 350 ms at Cz, consistent with RewP research using monetary reward tasks (Ethridge et al., 2017). We calculated unstandardized residual RewP to acceptance adjusting for RewP to rejection for analysis (Meyer et al., 2017). More positive values indicate greater responses to social reward. The RewP residual score has been shown to be reliably measured in this task (Ethridge and Weinberg, 2018).

### Data Analysis

To examine the associations between variables, bivariate correlation analyses were first conducted between residual RewP to social reward (i.e., RewP to acceptance adjusting for responses to rejection), clinical symptoms (depression, social anxiety), and social and non-social lifetime stress exposure. Next, both simple mediation and moderation analyses were conducted to examine the extent to which social reward responsiveness (residual RewP) mediated or moderated relationships between social and non-social stress exposure, and participants’ depressive and anxiety symptoms. To conduct these analyses, the PROCESS v3.1 macro for SPSS was used (Hayes, 2017).

## Results

### Preliminary Analyses

Participants’ lifetime social stress severity scores ranged from 0 to 71 (*M* = 25.62, *SD* = 15.59). Lifetime non-social stress scores ranged from 0 to 46 (*M* = 9.81, *SD* = 8.54). Participants’ depression scores (i.e., IDAS dysphoria symptoms) ranged from 10 to 42 out of a possible 50 (*M* = 21.86, *SD* = 7.51). Participants’ social anxiety scores ranged from 6 to 30 out of a possible 30 (*M* = 13.18, *SD* = 5.55). The IDAS dysphoria and social anxiety subscales had high internal consistency (Cronbach’s αs = 0.86 for each measure). With clinical cutoffs for IDAS identified by Stasik-O’Brien et al. (2018), 21.6% of participants were in the clinical range for symptoms of depression (clinical cut-offs for the social anxiety scale from the 99-item IDAS were not available).

**FIGURE 1:**
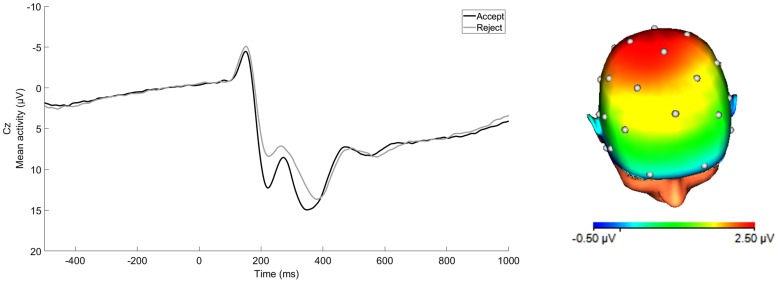
ERP waveform at Cz and scalp distribution at the 250–350 ms time window for average response to acceptance and rejection feedback corresponding to RewP. Scalp distribution reflects the response to acceptance minus rejection difference score. (32-channel montage with linked mastoid reference.)

ERP waveforms for acceptance and rejection conditions and corresponding scalp distribution for the difference of acceptance minus rejection conditions are presented in Figure 1. RewP to acceptance and rejection feedback had high split-half reliability at Cz (Spearman-Brown coefficients = 0.87 and 0.86, respectively). To examine associations between participants’ symptoms, social and non-social lifetime stress exposure, and residual RewP, bivariate correlation analyses were first conducted (see Table 1). As expected, greater lifetime social stress exposure was positively associated with depression (*r* = 0.37, *p* < 0.001) and social anxiety (*r* = 0.26, *p* < 0.001). Non-social stress exposure was also positively correlated with symptoms of depression (*r* = 0.38, *p* < 0.001) and social anxiety (*r* = 0.20, *p* = 0.002). Social and non-social stress exposure were not significantly correlated with the RewP residual score. The RewP residual score was not significantly correlated with either social anxiety or depressive symptoms, suggesting that social reward responsiveness did not mediate the association between lifetime stress exposure and participants’ symptom levels. Indeed, bootstrapped confidence intervals of tests of indirect effects of social and non-social lifetime stress exposure on internalizing symptoms through residual RewP all included 0 (see Table 2).

**TABLE 1:** Bivariate correlations between clinical symptoms, life stress variables, and social reward responsiveness.

**Variables**	***M* (*SD*)**	**1**	**2**	**3**	**4**	**5**
1. Depression	21.86 (7.51)	–				
2. Social anxiety	13.18 (5.55)	0.62^∗∗^	–			
3. Residual RewP	0.00 (3.83)	–0.10	–0.09	–		
4. Lifetime social stress severity	25.62 (15.59)	0.37^∗∗^	0.26^∗∗^	–0.04	–	
5. Lifetime non-social stress severity	9.81 (8.54)	0.38^∗∗^	0.20^*^	–0.04	0.57^∗∗^	–

^*^p < 0.01, ^∗∗^p < 0.001; RewP, Reward positivity.

**TABLE 2:** Model coefficients for simple mediation models testing effects of lifetime social and non-social stress severity and residual RewP on clinical symptoms.

	**Consequent**			
	
	**M (Residual RewP)**		**Y (Depression)**	
		
**Antecedent**	***b***	***SE***	***b***	***SE***
X (Social stress severity)	−0.01	0.02	0.18^∗∗^	0.03
M (Residual RewP)	–	–	−0.17	0.12
Constant	0.26	0.49	170.31^∗∗^	0.88
	*R*^2^ = 0.00, *F*(1, 229) = 0.40		*R*^2^ = 0.15, *F*(2, 228) = 19.57^∗∗^	
X (Non-social stress severity)	−0.02	0.03	0.33^∗∗^	0.05
M (Residual RewP)	–	–	−0.17	0.12
Constant	0.18	0.38	18.63^∗∗^	0.70
	*R*^2^ = 0.00, *F*(1, 229) = 0.40		*R*^2^ = 0.15, *F*(2, 228) = 20.33^∗∗^	
X (Social stress severity)	−0.01	0.02	0.09^∗∗^	0.02
M (Residual RewP)	*–*	*–*	−0.12	0.09
Constant	0.26	0.49	10.83^∗∗^	0.68
	*R*^2^ = 0.00, *F*(1, 229) = 0.40		*R*^2^ = 0.07, *F*(2, 228) = 9.18^∗∗^	
X (Non-social stress severity)	−0.02	0.03	0.13^*^	0.04
M (Residual RewP)	*–*	*–*	−0.12	0.09
Constant	0.18	0.38	11.91^∗∗^	0.55
	*R*^2^ = 0.00, *F*(1, 229) = 0.40		*R*^2^ = 0.05, *F*(2, 228) = 5.79^*^	

^*^p < 0.01, ^∗∗^p < 0.001; RewP, Reward positivity; b, unstandardized regression coefficients; SE, standard error.

### Moderation Analyses

Four moderation analyses were conducted to investigate relationships between social and non-social lifetime stress exposure, RewP residual scores, and depressive and anxiety symptoms. Specifically, we examined residual RewP as a moderator of associations between social and non-social lifetime stress exposure and depressive and social anxiety symptoms. Main effects of social stress or non-social stress and residual RewP were entered into each model. Then the interaction between either social or non-social stress and residual RewP was entered (see Table 3).

**TABLE 3:** Regression analyses testing the main and interaction effects of lifetime social and non-social stress severity and residual RewP on depressive symptoms (IDAS dysphoria subscale).

**Depressive Symptoms**		
**Lifetime Social Stress Severity**	**Unstandardized *b (SE)***	***p***

Social stress severity	0.18 (0.03)	<0.001
Residual RewP	0.24 (0.22)	0.268
Social stress severity X residual RewP	−0.02 (0.01)	0.024
	Change *R*^2^ = 0.02, *F*(1,227) = 5.19	
Total model	*R*^2^ = 0.17, *F*(3,227) = 15.01	<0.001

**Lifetime Non-social Stress Severity**	**Unstandardized *b (SE)***	***p***

Non-social stress severity	0.33 (0.05)	<0.001
Residual RewP	−0.02 (0.18)	0.929
Non-social stress severity X residual RewP	−0.02 (0.01)	0.251
	Change *R*^2^ = 0.01, *F*(1,227) = 1.33	
Total model	*R*^2^ = 0.16, *F*(3,227) = 14.01	<0.001

**Social Anxiety Symptoms**		

**Lifetime Social Stress Severity**	**Unstandardized *b (SE)***	***p***

Social stress severity	0.09 (0.02)	<0.001
Residual RewP	0.07 (0.17)	0.695
Social stress severity X residual RewP	−0.01 (0.01)	0.188
	Change *R*^2^ = 0.01, *F*(1,227) = 1.74	
Total model	*R*^2^ = 0.08, *F*(3,227) = 6.72	<0.001

**Lifetime Non-social Stress Severity**	**Unstandardized *b (SE)***	***p***

Non-social stress severity	0.13 (0.04)	0.00
Residual RewP	−0.10 (0.14)	0.502
Non-social stress severity X residual RewP	−0.00 (0.01)	0.791
	Change *R*^2^ = 0.00, *F*(1,227) = 0.07	
Total model	*R*^2^ = 0.05, *F*(3,227) = 3.86	0.010

*RewP = Reward positivity.*

**FIGURE 2:**
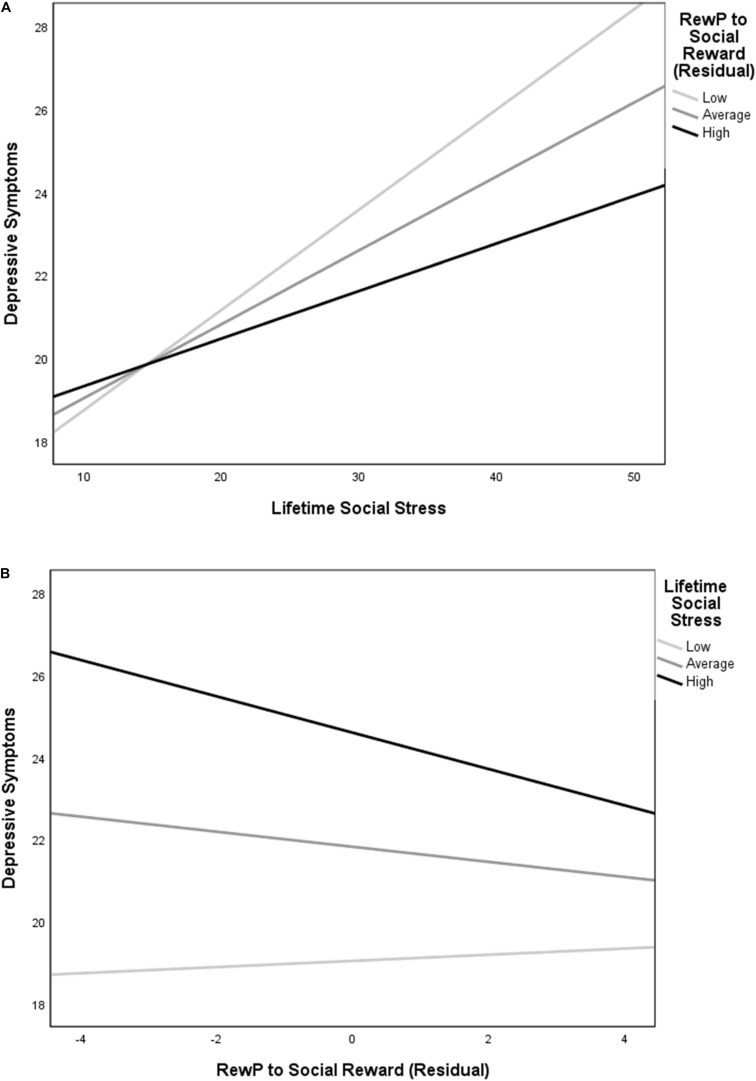
Simple slopes depicting **(A)** the relationship between social stress exposure and depression at low (-1 SD), mean, and high (+1 SD) residual RewP to social reward, and **(B)** the relationship between residual RewP to social reward and depression at low (-1 SD), mean, and high (+1 SD) social stress. Lifetime social stress exposure was positively associated with symptoms of depression at all levels of RewP, but with a relatively stronger magnitude of association at low compared to mean and high levels of residual RewP. Reduced RewP residual predicted more depressive symptoms only at a high level of social stress.

The overall model for lifetime social stress exposure predicting depressive symptoms was significant, *R*^2^ = 0.17, *F*(3, 227) = 15.01, *p* < 0.001. The significant main effect of social stress exposure in predicting symptoms of depression was qualified by an interaction between social stress exposure and RewP residual scores (see Figure 2A), *t*(227) = -2.28, *p* = 0.024. Decomposing this interaction using simple slopes revealed that greater lifetime social stress exposure predicted more depressive symptoms at low (-1 SD), mean, and high (+1 SD) levels of residual RewP. The magnitude of the relationship between social stress and depression was relatively stronger at low [simple slope = 0.24, *SE* = 0.04, *t*(227) = 5.96, *p* < 0.001] as compared to mean [simple slope = 0.18, *SE* = 0.03, *t*(227) = 6.10, *p* < 0.001], and high levels of residual RewP [simple slope = 0.11, *SE* = 0.04, *t*(227) = 2.85, *p* = 0.005]. To further understand this relationship, we also examined the effects of RewP at high and low levels of social stress. A reduced residual RewP predicted more depressive symptoms only at a high (+1 SD) level of social stress exposure [simple slope = -0.44, *SE* = 0.17, *t*(227) = -2.64, *p* = 0.009]. The simple slopes at low (-1 SD) and mean levels of social stress exposure were not significant (*ps* = 0.642 and 0.125, respectively; see Figure 2B).

**FIGURE 3:**
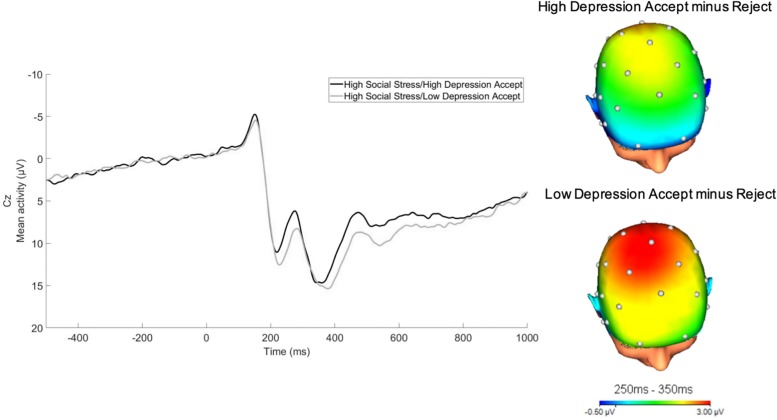
RewP to acceptance feedback at Cz in participants who experienced high lifetime social stress exposure (top 1/3). For illustrative purposes, a median split was computed to depict participants at high vs. low levels of depressive symptoms as measured by the IDAS dysphoria subscale following social stress exposure (32-channel montage with linked mastoid reference).

For illustrative purposes, we divided the social stress variable into thirds. We then split these participants based on their depressive symptoms into high and low depressive symptom groups via a median split. As depicted in Figure 3, RewP was relatively reduced in the high lifetime social stress exposure/high depressive symptom group as compared to the high lifetime social stress exposure/low depression group.

The overall models for lifetime social stress exposure predicting social anxiety symptoms, non-social stress exposure predicting depressive symptoms, and non-social stress exposure predicting social anxiety symptoms were all significant (see Table 3). However, only social and non-social lifetime stress exposure were significant predictors of clinical symptoms. The interactions between social or non-social stress exposure and participants’ residual RewP were not significant in these models, suggesting that residual RewP may be a relatively specific moderator of the impact of lifetime social stress exposure on symptoms of depression rather than social anxiety.

## Discussion

The present study examined associations between social and non-social lifetime stress exposure, social reward responsiveness as measured by RewP using the Island Getaway task, and symptoms of depression and social anxiety in a sample of emerging adults. Both social and non-social stress exposure were related to depressive symptoms. Additionally, social and non-social stress exposure were associated with social anxiety symptoms. In contrast, we did not find significant bivariate associations between the RewP residual score and participants’ symptoms, and results did not support social reward responsiveness (as measured by RewP) as a mediator of the effect of lifetime stress exposure on symptom levels. Instead, a significant interaction emerged between social stress and RewP to acceptance (adjusting for RewP to rejection via residual score) predicting depressive symptoms, such that the combination of greater lifetime social stress exposure and a reduced RewP to social reward was associated with greater depressive symptoms. Moreover, social reward responsiveness only predicted depressive symptoms at high levels of social stress. Finally, this moderation effect of RewP on symptom outcomes was unique to symptoms of depression and did not extend to symptoms of social anxiety.

Although preliminary and in need of replication, these results suggest that reduced social reward responsiveness may constitute a vulnerability for depressive symptoms following exposure to social stress, specifically. It is also possible that having greater reward responsiveness to social reward may help to protect against the impact of stress, particularly stress in interpersonal relationships. Individuals with blunted social reward responsiveness may be less likely to seek out and benefit from positive social interactions, which could inhibit their ability to cope with stress (Setterfield et al., 2016). As such, RewP to social feedback might predict more specific depressive symptom presentations, such as social withdrawal or social anhedonia. As RewP is relatively stable throughout development (Kujawa et al., 2018), identifying these specific symptom manifestations may improve understanding of depression onset and potential avenues for intervention before symptoms manifest (i.e., examining reduced social reward responsiveness and targeting these alterations early on).

More broadly, these findings emphasize the importance of examining social reward, in addition to monetary reward, in developmental trajectories of depression. Additional research should be conducted examining responses to multiple types of reward, including social reward, within the same sample to investigate whether particular types of reward responsiveness have unique predictive utility for depression. In addition, the current results emphasize the importance of considering specific dimensions of stressful experiences in clinical neuroscience research. That is, despite growing evidence that alterations in neural systems involved in positive emotions likely reflect a vulnerability that increase risk for later depression (for a review, see Kujawa and Burkhouse, 2017), little research has examined the possibility that a specific neural process might predict responses to specific types of stress. Despite the exploratory nature of the scoring of social and non-social stress scales used herein, the present study has taken a preliminary step to fill this gap.

Our results are broadly consistent with prior research showing that reduced activity in brain regions involved in reward processing may pose a potential increased risk for the development of depression in individuals exposed to stress (e.g., Nikolova et al., 2012; Corral-Frías et al., 2015). Despite a growing body of literature on the effects of stress and monetary reward responsiveness on depression, the present study is among the first to examine the effects of both life stress exposure and social reward responsiveness on depressive symptoms, and is the first to examine *lifetime* stress exposure. Our results suggest that, rather than directly explaining the relationship between life stress and depressive symptoms, reduced responsiveness to social reward may be a vulnerability factor specifically when people are exposed to social stress, a key risk factor for the development of depression. This suggests that individual differences in social reward responsiveness may be one factor that influences likelihood of developing depression following exposure to social stress, and, as such, individuals low in social reward responsiveness might benefit from targeted prevention.

Strengths of the current study include evaluation of competing hypotheses with regard to reward responsiveness as a mediator or moderator of the effects of stress on psychiatric symptoms, extension to the social reward domain, assessment of lifetime stress exposure, and tests of specificity of associations for depression or internalizing symptoms more broadly. A few limitations should be considered when interpreting these results. First, the study design was cross-sectional. For this reason, causality and the directionality cannot be determined. In particular, although mediation analyses can be performed with cross-sectional data, results should be replicated with longitudinal data. Second, given prior work linking reduced RewP and activation of ventral striatum to monetary reward to the later emergence of depressive symptoms (Kujawa and Burkhouse, 2017; Keren et al., 2018), we interpreted RewP as an indicator of a potential vulnerability for depression in the context of lifetime social stress exposure. However, the study design did not enable us to examine whether reduced RewP to social reward emerges prior to exposure to social stress or to increases in symptoms of depression. Future longitudinal research must be conducted to examine associations between social reward responsiveness and stress exposure across time and development, and to assess social reward responsiveness across levels of analysis, including behavior and circuit measures (National Institute of Mental Health [NIMH], 2019). Third, although the items that comprised the STRAIN social and non-social stress subscales were binned based on whether they were related to social situations or interactions, the present study is limited in its ability to test the validity of this scoring approach. The analyses of social vs. non-social stress scales of the STRAIN are exploratory and should be interpreted as such. Further work examining the extent to which these subscales converge with other indicators of social and non-social strain is needed. Fourth, although subthreshold depressive symptoms are a strong predictor of subsequently developing MDD (e.g., Keenan et al., 2008), this was a non-clinical sample and future research is needed to examine whether the present results generalize to clinical populations. Likewise, future studies could sample adolescents and adults from the community who have greater lifetime stress exposure burdens to examine the associations described here in other, more generally representative, populations. Fifth, we employed a self-report measure of current depression and anxiety symptoms in the present study, and it will be important for future studies to utilize interview-based assessments of participants’ symptoms and current and past history. Finally, given the number of models tested and relatively modest effect sizes, the current results must be interpreted cautiously, and replication is needed.

It is also worth noting that we only measured responses to social acceptance and rejection feedback, as opposed to neutral feedback for a few reasons. First, measuring acceptance/social reward and rejection feedback is consistent with a commonly used monetary reward paradigm to elicit RewP (Proudfit, 2015). In this task, the relative response to reward vs. loss has consistently been linked cross-sectionally and prospectively with depressive symptoms (Bress et al., 2015; Nelson et al., 2016; Kujawa et al., 2019). Second, evidence suggests that RewP presents as a relative positivity to monetary reward or the best possible outcome in a task and is less sensitive to differences between neutral and loss feedback (e.g., Kujawa et al., 2013). Finally, given the nature of social interaction tasks, “neutral” feedback is difficult to manipulate, as there would likely be individual differences in how people process feedback that is more ambiguous. The inclusion of a third condition would lengthen the task considerably. Nonetheless, additional research is needed to examine neural responses to neutral feedback in social vs. monetary reward tasks.

Notwithstanding these limitations, the present study is the first to examine how social reward processing is associated with lifetime stress exposure and depression and anxiety symptoms in a large sample of emerging adults – a developmental period when rates of depression increase dramatically (Kessler et al., 2001). The results highlight the potential utility of ERP measures of social reward responsiveness for clarifying pathways to the emergence of depression. In addition, they elucidate a pathway that appears to be relatively specific for lifetime social (vs. non-social) stress exposure in predicting depressive (vs. anxiety) symptoms. These findings may thus have implications for designing preventions targeting those low in social reward responsiveness, with the possibility of buffering against the negative effects of social stress before symptoms emerge.

## Data Availability

All datasets generated for this study are included in the manuscript and/or the supplementary files (10.6084/m9.figshare.9033842).

## Ethics Statement

This study was carried out in accordance with the recommendations of Declaration of Helsinki with written informed consent from all participants. All participants gave written informed consent in accordance with the Declaration of Helsinki. The protocol was approved b the McGill University research ethics board.

## Author Contributions

SP, PE, AW, and AK contributed to the design of this research study. PE and AW oversaw the conduct of the research study, data collection, and management. AK designed the original Island Getaway task. GMS created the STRAIN and oversaw the preparation of the stress data for inclusion, which was led by GSS. SP and AK analyzed and interpreted the data by consulting with all co-authors. SP and AK drafted the manuscript, which was subsequently revised by all co-authors. All authors read and approved the final version of the manuscript.

## Conflict of Interest Statement

The authors declare that the research was conducted in the absence of any commercial or financial relationships that could be construed as a potential conflict of interest. The reviewer JK declared a past collaboration with one of the authors AW to the handling Editor.
